# Revision Surgery Using Retrograde Nail versus Replating in Nonunion Distal Femur Fracture Treated with Plate

**DOI:** 10.1155/2022/5742743

**Published:** 2022-06-02

**Authors:** Antonio Ziranu, Giovanni Noia, Valerio Cipolloni, Michele Coviello, Giuseppe Maccagnano, Francesco Liuzza, Giulio Maccauro, Luigi Aurelio Nasto, Enrico Pola

**Affiliations:** ^1^Fondazione Policlinico Universitario A. Gemelli IRCCS, Rome 00168, Italy; ^2^Department of Clinical and Experimental Medicine, Azienda Ospedaliero Universitaria Ospedali Riuniti di Foggia, Foggia 71122, Italy; ^3^Department of Basic Medical Science, Neuroscience and Sensory Organs, Azienda Ospedaliero Universitaria Consorziale Policlinico, Bari 70124, Italy; ^4^Multidisciplinary Department of Medical-Surgical and Dental Specialties, University of Campania “Luigi Vanvitelli” School of Medicine, Naples 80138, Italy

## Abstract

Articular distal femur fractures represent 4% to 6% of femur fractures. Locking compression plates (LCPs) are the main treatment option. Nevertheless, a reoperation rate of 12.9% has been reported; nonunion is reported at 4.8%, delayed union at 1.6%, and malunion at 0.6%. Treatment of nonunions can be challenging as no unanimous consensus regarding the best surgical technique has been reached. The aim of this study was to evaluate and compare two types of revision surgery as treatment of LCP-treated articular distal femoral fracture nonunion: retrograde nail or replating. A retrospective cohort study of patients admitted from January 2015 to February 2017 for nonunion of AO/OTA 33C2 fractures previously treated with a lateral LCP was conducted. Patients were treated either with intramedullary nailing (Group A) or with replating (Group B). One independent observer performed clinically and radiographically followed up at 1, 3, 6, 9, 12, 24, and 36 months after surgery. The nonunion scoring system (NUSS) was used. Nine patients were included in our study. The mean follow-up was 2 years. Five patients were treated with intramedullary nailing and four with replating. The NUSS score was 24.2 ± 6.8 in the nailing group and 37.3 ± 3 in the replating group (*P*=0.03). In the nailing group, radiographic consolidation was obtained in all cases. In the replating group, nonunion was found in 3 patients and failure of osteosynthesis in one patient. Therefore, four patients (Group B) underwent implant removal and retrograde femoral nailing, obtaining radiological healing. The union time was 7.6 months in the nailing group. Retrograde intramedullary nailing can be used as an effective treatment of aseptic AO-33C distal femoral nonunion following primary locking plating.

## 1. Introduction

Distal femur fractures (DFFs) represent 4% to 6% of all femur fractures [1, 2]. Different treatments have been proposed for these fractures, such as retrograde intramedullary nailing, open reduction, and internal fixation with condylar locking compression plates (LCPs) or distal femoral replacement [3, 4]. Locking plates are often indicated because of their capability to provide high stability and to restore articular surface [5]. Despite their many advantages, locking plates are not without complications. Implant failure, nonunion, delayed union, and malalignment have been reported by several authors [6]. A recent meta-analysis [7] reports a reoperation rate for DFF of 12.9%; nonunion is reported in 4.8% of patients, mechanical failure in 3.6%, delayed union in 1.6%, and malunion in 0.6%.

DFF with articular involvement is notoriously difficult to treat. A perfect reconstruction of the articular surface anatomy, restoration of alignment and rotation, and a stable synthesis, often obtained through the use of an LCP, are required [3]. When an aseptic nonunion occurs in articular fractures, previously treated with LCP, the revision treatment can become a challenge.

Nonunion fracture factors include patients' comorbidities such as diabetes and vascular disease and factors which influence patients' immune response such as advanced age, smoking, malignancy, rheumatoid disease, NSAID use, and steroid use [7]. Various techniques have been described to treat nonunions including replating [8], intramedullary nailing [8, 9], fixed angle plating with bone grafting [10, 11], combined lateral and medial plating [12], and combined nail, plate, and autologous graft positioning [13].

A thorough classification of nonunions is mandatory to correctly plan the treatment. Various classification scores have been proposed to aid the surgeon in the decisional process [14–16]. The nonunion scoring system (NUSS) is a validated score that incorporates a treatment algorithm [16, 17]. It analyses general and local risk factors and identifies mechanical and biological issues, classifying patients into categories based on these parameters and suggesting the appropriate treatment.

There is no unanimous consensus regarding the technique that should be used as a treatment of aseptic articular femoral nonunions following locked plating.

The aim of this study was to compare clinical and radiological outcomes of DFF nonunions treated with retrograde locked nailing vs. LCP replating.

## 2. Materials and Methods

### 2.1. Design

Following Institutional Review Board (IRB) approval (protocol code ORT2021_04, approved on 15 February 2021), our institutional database was searched for patients admitted to the Traumatology Unit of our institution from January 2015 to February 2017.The inclusion criteria for the study were as follows: (1) 33C2 closed fractures (AO/OTA classification), (2) patients treated with a lateral locking plate, (3) nonunion after treatment, and (4) age over 18 years old. The exclusion criteria were as follows: (1) pathological fractures, (2) infection of the surgical site, (3) osteomyelitis, and (4) absence of a new postoperative traumatic event.

### 2.2. Settings and Participants

Between January 2015 and February 2017, fifteen patients with a distal femur fracture were surgically treated in our clinic with LCP and were evaluated for eligibility for the present study. Five patients did not meet the inclusion criteria, and one patient died one month after surgery. With those six patients excluded, nine patients who met the inclusion criteria were included in the study. A diagnosis of nonunion was made when at least 9 months had elapsed from fracture and no visible signs of healing were visible for at least 3 months [16]. After revision surgery, all patients were then divided into two groups according to the choice of surgery treatment: patients who had been treated with retrograde locked nailing were assigned to Group A and those who managed with replating had been assigned to Group B. All patients were treated by the same surgical team with more than seven years of experience in lower limb surgery (AM and AZ). No patient died during the study.

### 2.3. Data Measurement

For each patient, age, gender, mechanism of injury, AO/OTA classification of the fracture, NUSS [18] score, implant used in the primary surgery, postoperative complications, follow-up length, union time (UT), range of motion (ROM), and Neer score [19] at final follow-up were collected.

### 2.4. Surgical Procedure

The revision surgery was performed in the supine position on a radiolucent table with the knee flexed (approximately 60°) to reduce the traction force of the gastrocnemius muscle. All screws and plates were removed, and a thorough debridement was performed according to the manufacturer's removal instructions.

A transpatellar tendon approach was used for Group A patients. The entry point in the intercondylar notch was identified, and a guide wire was inserted into the medullary canal. The medullary canal was reamed as widely as possible, and then, a retrograde nail was placed, according to the manufacturer's instructions. The distal fixation was chosen case by case. A static proximal fixation was used in every case.

Group B patients underwent revision surgery with replate. If fracture reduction was considered not acceptable, a new reduction was performed. A longer plate was positioned with a less invasive stabilization system (LISS) for distal femur LCP. No variable angle screws were used, and the plate was positioned according to the manufacturer's instructions. The LISS system allowed to avoid further damage to the soft tissues while positioning the proximal screws.

Union of the fracture was diagnosed when the patient had no local pain and tenderness and could walk without aids, and a solid callus connecting both fragments could be seen radiographically [20]. In both groups, if the NUSS score was >25, pulsed electromagnetic fields (PEMFs) were prescribed [21]. Postoperatively, patients were encouraged to perform the knee range of motion exercises and early non-weight-bearing ambulation with a walker assistance was allowed. After 4 weeks, protected weight-bearing was allowed. Patients were routinely followed up clinically and radiographically at one, three, six, nine, 12, 24, and 36 months after surgery by the same operator for each patient.

### 2.5. Statistical Analysis

All data were collected electronically and were analysed using Software SPSS 25.0 (IBM, Armonk, NY, USA). In order to account for nonnormality (Shapiro–Wilk test), continuous variables were reported as the mean and standard deviation and compared (univariable analysis) with the Mann–Whitney *U* test. Categorical variables were reported as absolute and relative frequencies and compared (univariable analysis) with the Spearman correlation test. Statistical significance “alpha” was fixed to 0.05. The primary endpoint examined was the NUSS score. The secondary endpoint was clinical and knee functionality.

## 3. Results

Nine patients with AO 33-C2 fracture were included in our study. The primary injury was due to a traffic accident in seven patients and to fall from height in two patients. All patients received first treatment with an LCP and developed nonunion of the fracture. We reported the significant data (Table 1).

**Figure 1:**
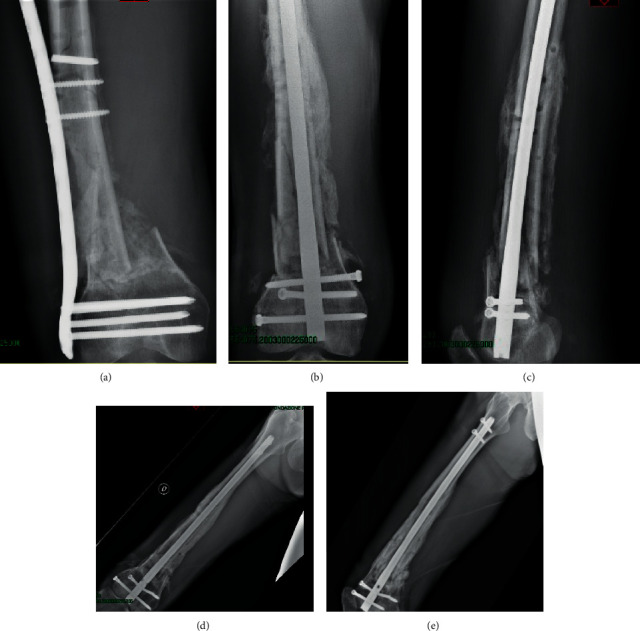
(a) Preoperative nonunion. (b, c) Two-month postoperative nonunion treated with a retrograde nail. (d, e) Twelve-month postoperative.

**Figure 2:**
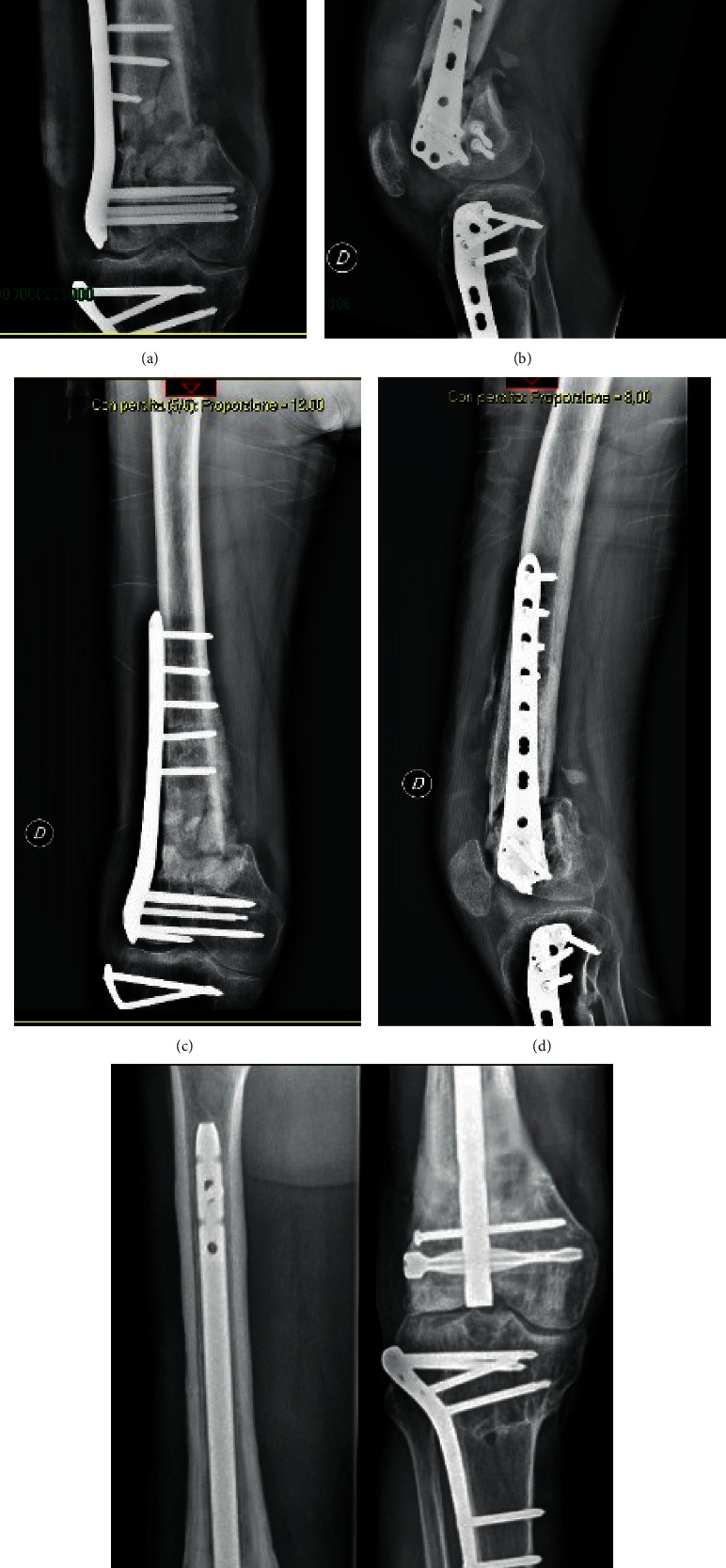
(a, b) Preoperative nonunion. (c, d) Six-month postoperative nonunion treated with replating with a new nonunion. (e) Twelve-month postoperative nonunion of the replating treated with a retrograde nail.

Five patients, two females (40%), were treated with intramedullary nailing (Group A), and four patients, two females (50%), were treated with replating (Group B). No significant differences were found between the groups and the sex or type of injury analysing data with the Spearman correlation test. The mean age was 47.6 ± 7.9 years old in the nailing group and 56 ± 7.2 years old in the replating group (*P*=0.11). The average NUSS score at the sixth month was 24.2 ± 6.8 in the nailing group and 25.5 ± 7.6 in the replating group (*P*=0.73). The group treated with retrograde femoral nailing presented radiographic consolidation in all cases (Figures 1(a)–1(e). The replating group experienced nonunion in 3 patients and failure of osteosynthesis in one patient. Therefore, all patients in the replating group underwent implant removal and retrograde femoral nailing, with radiological complete healing of the fracture (Figures 2(a)–2(e)). The average NUSS score in the replating group before retrograde nail positioning was 37.3 ± 3 (*P*=0.03 compared with the Group A NUSS score at the same time).

The average ROM at final follow-up was 100 ± 10° in the nailing group and 68.2 ± 7.8° in the replating group (*P*=0.02). The time to union was 7.6 ± 1.7 months in the nailing group, whereas no signs of union were observed in the replating group. After secondary treatment with nailing in this group, time to union was 9.3 ± 1.2 months (*P*=0.19). The mean value of the Neer score was 72.8 ± 6.4 (good) and 61.5 ± 4.3 (fair) in the nailing and the replating group, respectively (*P*=0.03). The mean follow-up was 2 years.

One superficial infection of the surgical wound was recorded in the replating group. One patient (20%) in the nailing group had a mobilization of the nail due to insufficient axial stiffness of the distal screws, so the patient underwent removal of the nail distal screws and spiral blade positioning (Table 2).

## 4. Discussion

The NUSS score represents a useful index for nonunion fracture treatment. Using this score, orthopaedic surgeons can divide patients into four groups: nonunions due to the main mechanical problem, nonunions with both minor mechanical and biological problems, nonunions with impairment of both biological and mechanical conditions (major mechanical and minor biological or minor mechanical and major biological problem), and the fourth group with both major biological and mechanical problems [16–18]. Correctly classifying the nonunion guides the surgeon to the appropriate treatment.

Delayed union and nonunion after locking plate fixation in DFF have been reported to occur in 1.6% and 4.5% of cases [7]. The treatment of these nonunions can be challenging even for experienced surgeons.

Intramedullary nailing, through various approaches, has proved to be an effective treatment in clinical and biomechanical studies of tibial [22, 23] and femoral [24] acute fractures and relative nonunion and could be considered as a valid alternative option especially in osteoporotic bone [25, 26].

No studies reported the treatment of aseptic nonunion of AO-33C2 DFF treated with the LCP. In the literature, several studies analysed the results of the treatment of nonunions of supracondylar femoral fractures (AO-33A2, A3) [20, 24, 26, 27]. A radiographical fracture healing in 100% of cases and good clinical results were achieved by Khan et al. [24] using retrograde intramedullary nailing for supracondylar femoral nonunions. Wu et al. [20] reported the results of intramedullary nailing for distal femur nonunions after plating in 18 patients. They achieved an 88.9% of union rate, satisfactory functional outcomes, and good limb alignment at 4.2 months of follow-up.

In a group of 21 patients followed up for 3.4 years, Wu et al. [27] obtained union in 4.3 months in all patients after intramedullary nailing of supracondylar femur fracture nonunion treated with plating. They achieved good ROM recovery but reported 3 malunions.

These encouraging results obtained with intramedullary nailing could be related to the recanalization of the marrow cavity, preservation of the periosteal blood supply, and greater stiffness in the axial load that allows early rehabilitation and weight-bearing [27].

Our patients suffered from nonunion of AO-33C2 fractures previously treated with the distal femur LCP. We compared two different techniques as revision surgery. Patients treated with retrograde intramedullary nailing had better postsurgical outcomes: in all patients, clinical and radiological healings were achieved with reduced knee stiffness (*P*=0.02 in ROM mean values), a more rapid consolidation of the nonunion (*P*=0.03 in NUSS mean values at last follow-up), and a more precocious segmental and global functional recovery compared to the replating group (*P*=0.03 in Neer mean values). There was only one complication in this group, that is, a pullout of a distal locking screw; in this case, the fracture healed after revision of the distal fixation. In the replating group, we recorded 3 cases of pseudarthrosis and 1 case of failure of the synthesis. In these patients, a new surgical revision was performed using a retrograde intramedullary nail, obtaining in all cases clinical and radiological healing, but a longer consolidation time and a lower rate recovery of knee motion.

The main limitation of the present study is the small sample size. On the other hand, the strengths of the study are the use of multiple subjective and objective functional scores, and it is one of the first studies in the literature analysing distal femur revision surgery with different devices.

Due to the small number of patients included and the retrospective nature of this study, we cannot pronounce against the use of replating in the treatment of articular distal femur nonunions, but our results require the need of further trials.

## 5. Conclusions

Distal femur fracture revision surgery represents a challenge for the surgeon. Although a consensus was not reached, according to our results, retrograde intramedullary nailing can be used as an effective treatment of aseptic AO-33C2 distal femoral nonunion following primary locking plating resulting in clinical and radiological healing with reduced knee stiffness.

## Figures and Tables

**Table 1:** Characteristics of the sample (*n* = 9).

	Main group	Group A	Group B
Type of injury			
Traffic accident, *n* (%)	7 (77.8%)	4 (56.2%)	3 (43.8%)
Fall from height, *n* (%)	2 (22.2%)	1 (50%)	1 (50%)

Gender			
Male, *n* (%)	5 (55.6%)	3 (60%)	2 (40%)
Female, *n* (%)	4 (44.4%)	2 (50%)	2 (50%)
Age, mean ± SD	51.3 ± 8.4	47.6 ± 7.9	56 ± 7.2

Side			
Left, *n* (%)	4 (44.4%)	1 (25%)	3 (75%)
Right, *n* (%)	5 (55.6%)	3 (60%)	2 (40%)

**Table 2:** Clinical and radiological outcomes at last follow-up according to the surgical treatment.

	Group A	Group B	*P* value
NUSS	21.2 ± 6.8	37.3 ± 3	0.03
ROM	100 ± 10°	68.2 ± 7.8°	0.02
Neer score	72.8 ± 6.4	61.5 ± 4.3	0.03
Union time (months)	7.6 ± 1.7	9.3 ± 1.2	0.19
Complication	1 (20%)	1 (25%)	0.24

NUSS: the nonunion scoring system.

## Data Availability

The data used to support the findings of this study are available from the corresponding author upon request.
